# Supplementary material for: Knockout or inhibition of USP30 protects dopaminergic neurons in a Parkinson’s disease mouse model
Source: Nat Commun. 2023 Nov 13;14:7295. doi: 10.1038/s41467-023-42876-1 (PMC10643470; doi:10.1038/s41467-023-42876-1)
Supplement: Supplementary file 1 — Supplementary Information [file 41467_2023_42876_MOESM1_ESM.pdf]

Knockout or inhibition of USP30 protects dopaminergic neurons in a Parkinson's disease mouse model

Authors: Tracy-Shi Zhang Fang[1]*, Yu Sun[2], Andrew C. Pearce[3], Simona C. Eleuteri[1], Mark Kemp[3], Christopher A. Luckhurst[3], Rachel Williams[3], Ross Mills[3], Sarah Almond[3], Laura Burzynski[3], Nóra M Márkus[3], Christopher J Lelliott[4], Natasha A Karp[4], David J. Adams[4], Stephen P. Jackson[3,5,6], Jin-Feng Zhao[7], Ian G. Ganley[7], Paul W. Thompson[3]*, Gabriel Balmus[2,8]*, David K. Simon[1]

Affiliations:

1. Department of Neurology, Beth Israel Deaconess Medical Center and Harvard Medical School; Boston, Massachusetts, United States of America.
2. UK Dementia Research Institute at the University of Cambridge and Department of Clinical Neurosciences, University of Cambridge, Cambridge Biomedical Campus, Cambridge, CB2 0AH, UK.
3. Mission Therapeutics Ltd. Glenn Berge Building, Babraham Research Campus, Cambridge CB22 3FH, UK.
4. Wellcome Sanger Institute, Cambridge, CB10 1SA, UK.
5. The Gurdon Institute and Department of Biochemistry, University of Cambridge, Cambridge, CB2 1QN, UK.
6. Cancer Research UK Cambridge Institute, University of Cambridge, Cambridge Biomedical Campus CB2 0RE, UK.
7. MRC Protein Phosphorylation and Ubiquitylation Unit, University of Dundee, Dundee, DD1 5EH, UK
8. Department of Molecular Neuroscience, Transylvanian Institute of Neuroscience, 400191, Cluj-Napoca, Romania

*Corresponding authors.

Email: szhang9@bidmc.harvard.edu, PThompson@missiontherapeutics.com, gb318@cam.ac.uk

One Sentence Summary:

Knockout or inhibition of USP30 promotes the degradation of dysfunctional mitochondria and is neuroprotective in an α-synuclein mouse model of Parkinson's.

Supplementary Figure 1. Graphical representation of the phenotyping with significant terms from Extended data 1. a, Dot plot ± s.d. of individual mice showing the white blood cell counts in WT (n=341 females; n=340 males), and *Usp30* KO mice (n=6 females; n=7 males) display mild sexual dimorphism (*p=0.04546; mixed model framework, linear mixed-effects model). **b,** Dot plot ± s.d. of individual mice showing the mean corpuscular haemoglobin volume WT (n=341 females; n=340 males), and *Usp30* KO mice (n=6 females; n=7 males) display mild sexual dimorphism (*p=0.04141; mixed model framework, linear mixed-effects model). **c,** Dot plot ± s.d. of individual mice shows that male *Usp30* KO mice (n=7) have increased alkaline phosphatase as compared to WT male mice (n=340) (*p=0.04046; mixed model framework, linear mixed-effects model). The alkaline phosphatase (unit/L) was measured from peripheral blood. No difference was detected in female mice. **d,** Analysis of plasma glucose concentration (mg/dl) following intraperitoneal challenge with 2g/kg glucose shows that male *Usp30* KO mice (n=7) have increased plasma glucose levels at 30 minutes post injection (***p= 0.00065; mixed model framework, linear mixed-effects model; error bars s.d.) but then recover and behave like WT mice (n=340). No difference was detected in female mice. No difference in the Area Under the Curve (AUC ± s.d.) was detected for either male WT (n=341 females; n=340 males), and *Usp30* KO mice (n=6 females; n=7 males). **e,** Dot plot ± s.d. of individual mice showing that the neutrophil number in WT (n=341 females; n=340 males), and *Usp30* KO mice (n=6 females; n=7 males) shows mild sexual dimorphism (*p=0.04565; mixed model framework, linear mixed-effects model). **f,** Dot plot ± s.d. of individual mice showing that the Cd4 Cd44 Cd62l Alpha Beta T cell number in WT (n=341 females; n=340 males), and *Usp30* KO mice (n=6 females; n=7 males) shows mild sexual dimorphism (*p=0.04222; mixed model framework, linear mixed-effects model). **g,** Dot plot ± s.d. of individual mice showing that the Ly6c positive monocyte number in WT (n=341 females; n=340 males), and *Usp30* KO mice (n=6 females; n=7 males) display mild sexual dimorphism (*p=0.04046; mixed model framework, linear mixed-effects model). Source data are provided as a Source Data file.

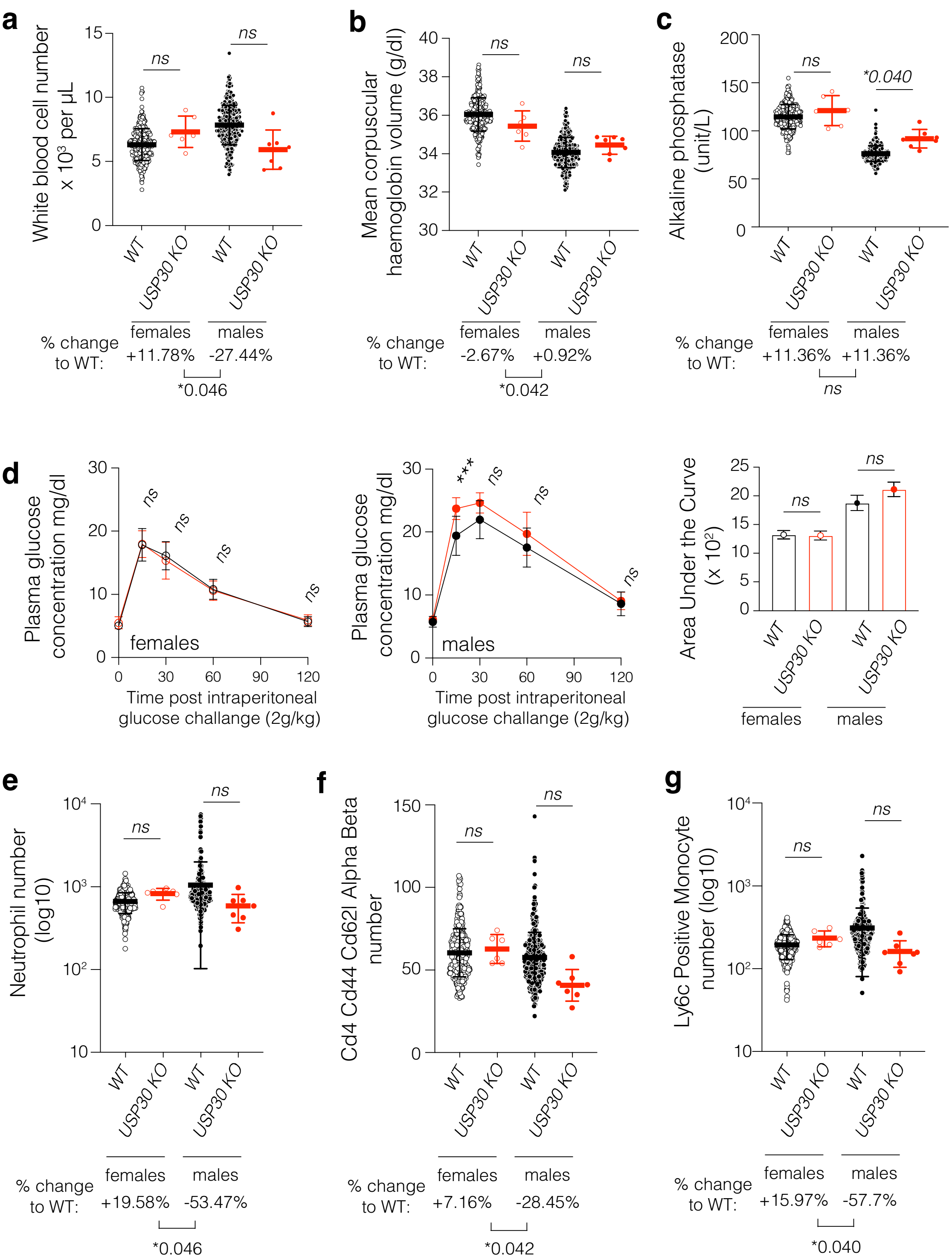
a
White blood cell number x 10³ per μL
ns
ns
WT
USP30 KO
WT
USP30 KO
% change to WT:
females
males
+11.78%
-27.44%
*0.046
b
Mean corpuscular haemoglobin volume (g/dl)
ns
ns
WT
USP30 KO
WT
USP30 KO
% change to WT:
females
males
-2.67%
+0.92%
*0.042
c
Alkaline phosphatase (unit/L)
ns
*0.040
WT
USP30 KO
WT
USP30 KO
% change to WT:
females
males
+11.36%
+11.36%
ns
d
Plasma glucose concentration mg/dl
females
Time post intraperitoneal glucose challange (2g/kg)
ns
ns
ns
ns
Plasma glucose concentration mg/dl
males
Time post intraperitoneal glucose challange (2g/kg)
***
ns
ns
ns
Area Under the Curve (x 10²)
ns
ns
WT
USP30 KO
WT
USP30 KO
females
males
e
Neutrophil number (log10)
ns
ns
WT
USP30 KO
WT
USP30 KO
% change to WT:
females
males
+19.58%
-53.47%
*0.046
f
Cd4 Cd44 Cd62l Alpha Beta number
ns
ns
WT
USP30 KO
WT
USP30 KO
% change to WT:
females
males
+7.16%
-28.45%
*0.042
g
Ly6c Positive Monocyte number (log10)
ns
ns
WT
USP30 KO
WT
USP30 KO
% change to WT:
females
males
+15.97%
-57.7%
*0.040

Supplementary Figure 2. *Usp30* KO mice show lower fat liver accumulation with ageing. a, Hematoxylin and eosin staining of livers from WT (left) and *Usp30* KO (right) one year old mice show that USP30 loss delays accumulation of fat in the liver. **b,** Dot plot of individual mice showing the quantification of the percentage fatty liver in WT *vs* *Usp30* KO mice. Significance determined by unpaired Mann-Whitney nonparametric test. Error bars represent mean ± s.d.; *, $P<0.05$. Inlets enlarged in lower panels. Scale bar, 1mm for upper panels, 10 μm for lower panels. Source data are provided as a Source Data file.

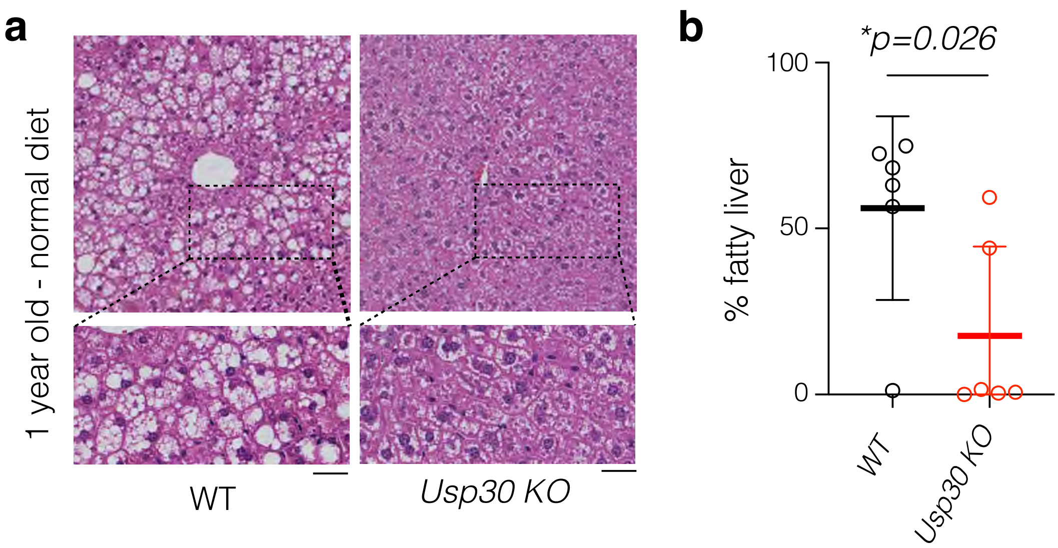

Supplementary Figure 3. Mitophagy signal in other tissues and brain regions of *Usp30* KO mice and WT mice. a, Representative fluorescence images show the mito-QC fluorescence signal (mCherry-red, GFP-green) in skeletal muscle and cardiac muscle from WT and *Usp30* KO female mice. Scale bar, 10μm. **b,** Quantification of mitolysosome area / mitochondrial area in skeletal muscle (left panel) and cardiac muscle (right panel) (n=8 mice per group). **c**, Representative fluorescence images show the mCherry, LAMP1 and NeuN signal in cortex and hippocampus of WT and *Usp30* KO male mice. Scale bar, 10 μm for left panels, 1 μm for enlarged inlets. **d**, Quantification of fluorescence intensity of mCherry puncta in the neurons in respective brain regions. (n=3 male mice per group, 5-10 neurons per mice). WT and *Usp30* KO in the bar graph represent *mito*-QC and *mito*-QC/Usp30 KO, respectively. Significance determined by unpaired, two-tailed Student's t-test. Error bars represent mean ± s.d.; * P<0.05. Source data are provided as a Source Data file.

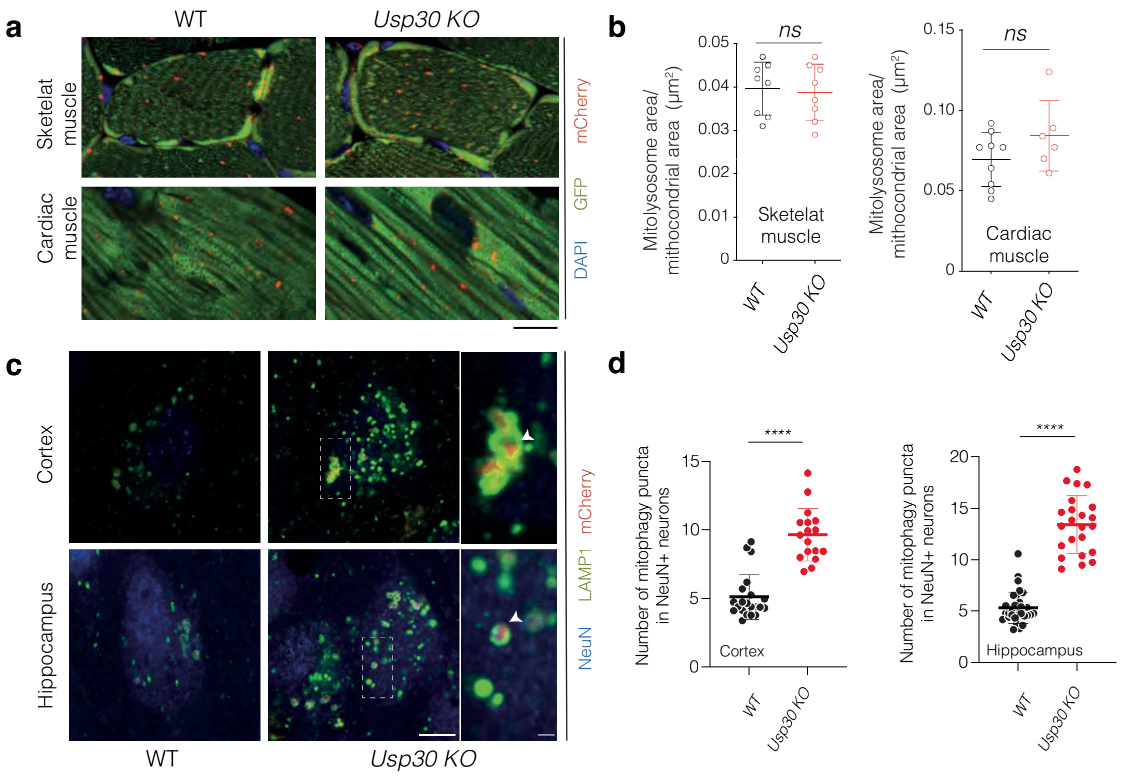

Supplementary Figure 4. *Usp30* KO increased mitophagy signal in DA neurons of SNpc regardless of αSyn overexpression. a, Representative fluorescence images show the OPA-1 fluorescence signal (red), the lysosomal marker, LAMP1 (green), and dopaminergic neurons (TH, blue) in the contralateral and ipsilateral SNpc of *mito*-QC and *mito*-QC/Usp30 KO male mice with AAV-SNCA injection. Dashed white lines indicate the boundaries of dopaminergic neurons. Scale bar, 10 μm. **b**, Quantification of colocalized *OPA-1* and LAMP1 puncta in individual dopaminergic neurons of the SNpc (n=3 mice per group, 5-10 neurons per mice). Each dot represents one dopaminergic neuron. WT and *Usp30* KO in the bar graph represent *mito*-QC and *mito*-QC/Usp30 KO, respectively. NI: no contralateral injection; **c,** Representative striatal sections of TH immunohistochemistry in male mice at 28 weeks post-injection of AAV-A53T-SNCA.; enlarged in lower panels. Scale bar, 1mm for upper panels, 100 μm for lower panels. Relative optical density of TH+ fibres in the ipsilateral striatum compared with the contralateral side of male mice in each group (n=5-8 mice per group). Significance determined by one-way ANOVA. Error bars represent mean ± s.d.; ****P<0.0001. Source data are provided as a Source Data file.

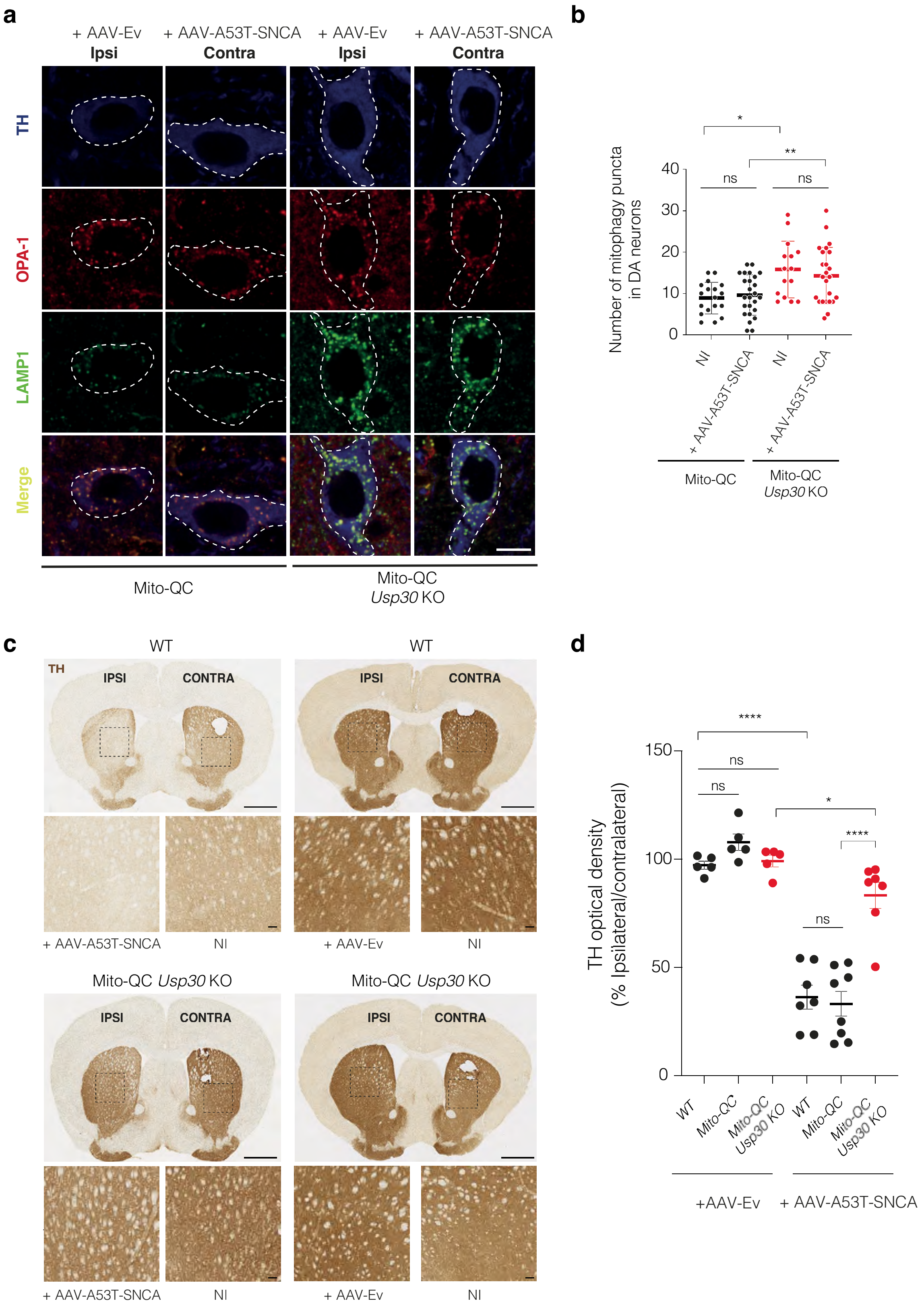

a
+ AAV-Ev Ipsi
+ AAV-A53T-SNCA Contra
+ AAV-Ev Ipsi
+ AAV-A53T-SNCA Contra
TH
OPA-1
LAMP1
Merge
Mito-QC
Mito-QC Usp30 KO
b
Number of mitophagy puncta in DA neurons
40
30
20
10
0
*
**
ns
ns
NI
+ AAV-A53T-SNCA
NI
+ AAV-A53T-SNCA
Mito-QC
Mito-QC Usp30 KO
c
WT
WT
TH
IPSI
CONTRA
IPSI
CONTRA
+ AAV-A53T-SNCA
NI
+ AAV-Ev
NI
Mito-QC Usp30 KO
Mito-QC Usp30 KO
IPSI
CONTRA
IPSI
CONTRA
+ AAV-A53T-SNCA
NI
+ AAV-Ev
NI
d
TH optical density (% Ipsilateral/contralateral)
150
100
50
0
****
ns
ns
*
****
ns
WT
Mito-QC
Mito-QC Usp30 KO
WT
Mito-QC
Mito-QC Usp30 KO
+AAV-Ev
+ AAV-A53T-SNCA

Supplementary Figure 5. a, USP30 depletion decreased αSyn pathology in the SNpc of the AAV-A53T-SNCA mouse model. **a,** Representative SNpc sections of TH (red) and phospho-S129 αSyn (S129, green) in different groups of male mice at 28 weeks post-injection of AAV-A53T-SNCA (used in Fig. 2c) or AAV-empty vectors. The inset of left panels is enlarged to the right panels. Scale bar, 1mm for left panels, 100 μm for right panels. **b**, Representative images shown immunofluorescent signals of mitochondrial marker, OPA-1 (red) and S129 phospho-αSyn (green) in the neurons of ipsilateral SNpc from AAV-SNCA male mice. **c**, Quantification of the fraction of OPA-1 overlaps with s129 phospho-αSyn in the neurons. **d**, Quantification of the fraction of s129 phospho-αSyn overlaps with OPA-1 in the neurons. (n=3 mice per group, 5-10 neurons per mice, one dot represents one neuron). Significance determined by one-way ANOVA. Error bars represent mean ± s.d.; * $P<0.05$, **** $P<0.0001$. Source data are provided as a Source Data file.

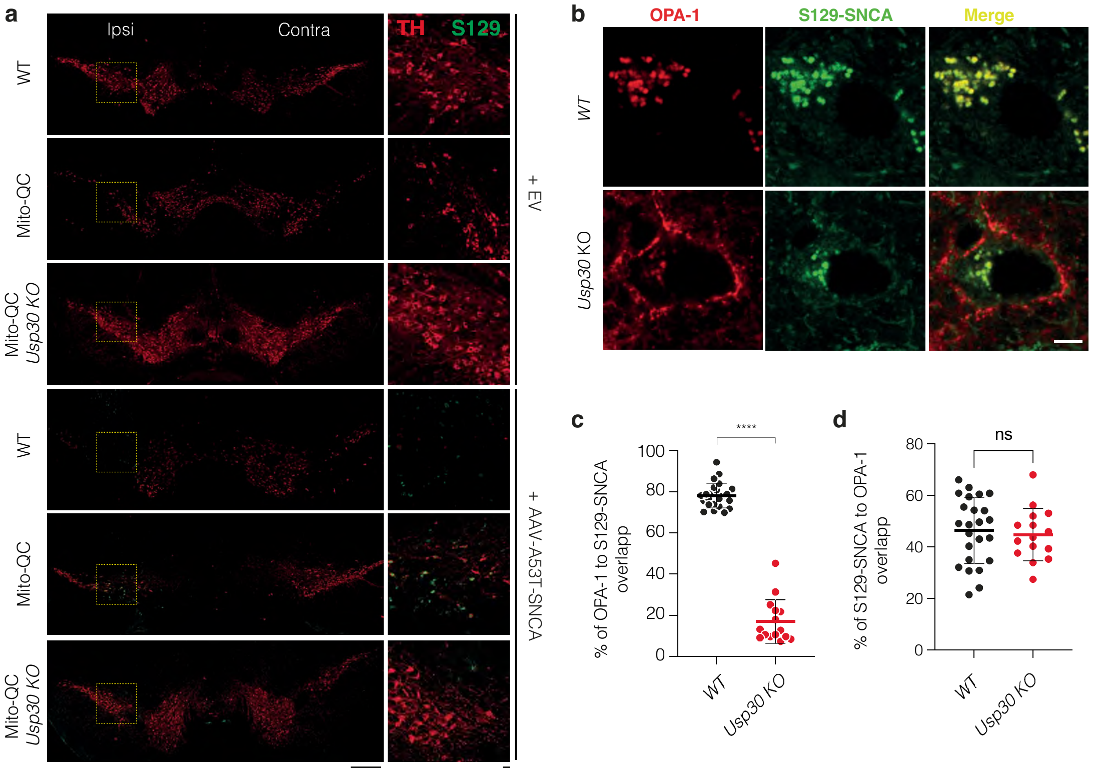

Supplementary Figure 6. Effects of USP30 inhibitor, MTX115325, on Ubiquitylated TOM20 in IPSC dopaminergic neurons. a, Key properties of USP30 inhibitor, MTX115325. b, Representative western blot images of TOM20, TOM20-ub, beta-actin from control IPSC dopaminergic neuron lysates after treatment with MTX115325. c, Quantitation of TOM20-ub band intensity after 24 hours DMSO as vehicle control or 24 hours MTX115325 (1 µM) treatment in WT control and A53T αSyn mutated dopaneurons. Densitometry data was normalised to TOM20 and relevant DMSO controls Significance was determined using Mann-Whitney test, * $P<0.05$. d, Quantitation of TOM20-ub band intensity after 7 day DMSO as vehicle control or 7 day MTX115325 (0.01, 0.1 or 1 µM) treatment in WT control and A53T αSyn mutated dopaneurons. Densitometry data was normalised to TOM20 and relevant DMSO controls Significance was determined using Kruskal-Wallis test with Dunn’s multiple comparison posthoc test * $P<0.05$, ** $P<0.01$ e, Graph shows brain USP30 engagement of MTX115325 after 10mg/kg PO dosing in male mice. Source data are provided as a Source Data file.

a

Key properties of the USP30i (MTX115325)

Assay	MTX115325 (USP30i)
Human USP30 FP assay (IC50)	12 nM
Cellular ubiquitin probe assay (IC50)	25 nM
TOM20-ub HeLa-PARKIN + A/O (EX1.5x; EC50)	10 nM ; 32 nM
Selectivity vs DUBs; Cathepsins (IC50)	24,926 nM (USP2); 42,082 nM (Cath L)
Mouse USP30 FP assay (IC50)	13 nM
Mouse oral bioavailability (%)	98%
Mouse clearance (blood mL/min/kg)	19.7
CNS penetration (Kpu,u; arithmetic mean)	0.4

b

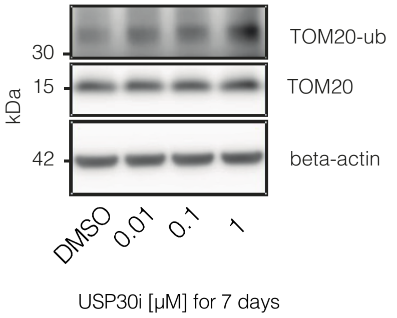

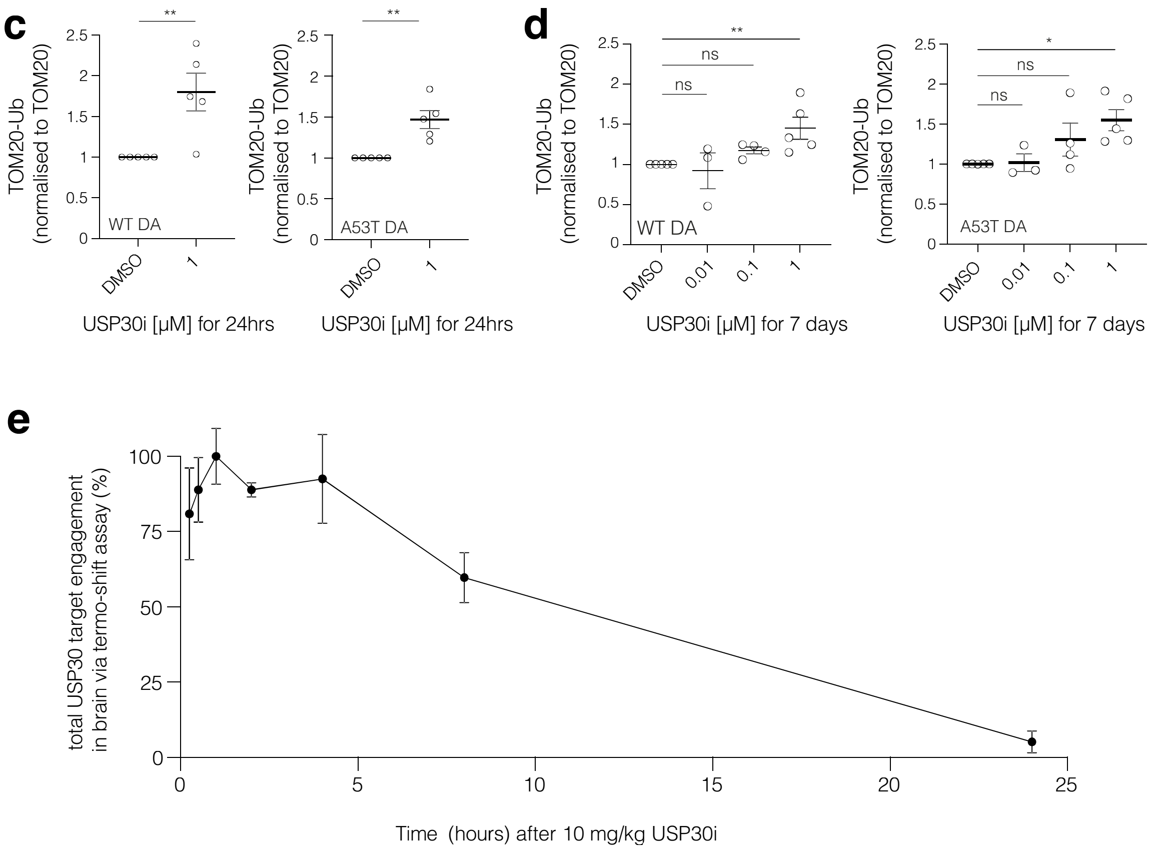

Supplementary Figure 7. USP30 inhibition leads to lower numbers of microglia and activated astrocytes. Percentage of Iba-1 staining **(a)**, GFAP staining **(b)**, αSyn staining **(c)** and number of Ser129-αSyn cells/mm2 **(d)**, in SNpc area in the non-injected (NI) side versus the AAV-A53T-SNCA injected side in USP30 inhibitor treated versus vehicle treated animals. n>10 mice per group. Significance determined by using T tests between vehicle and MTX115325 treated groups for each separate endpoint. Scale bar 100 μm. ns, not significant; error bars s.d.; *P<0.05. Source data are provided as a Source Data file.

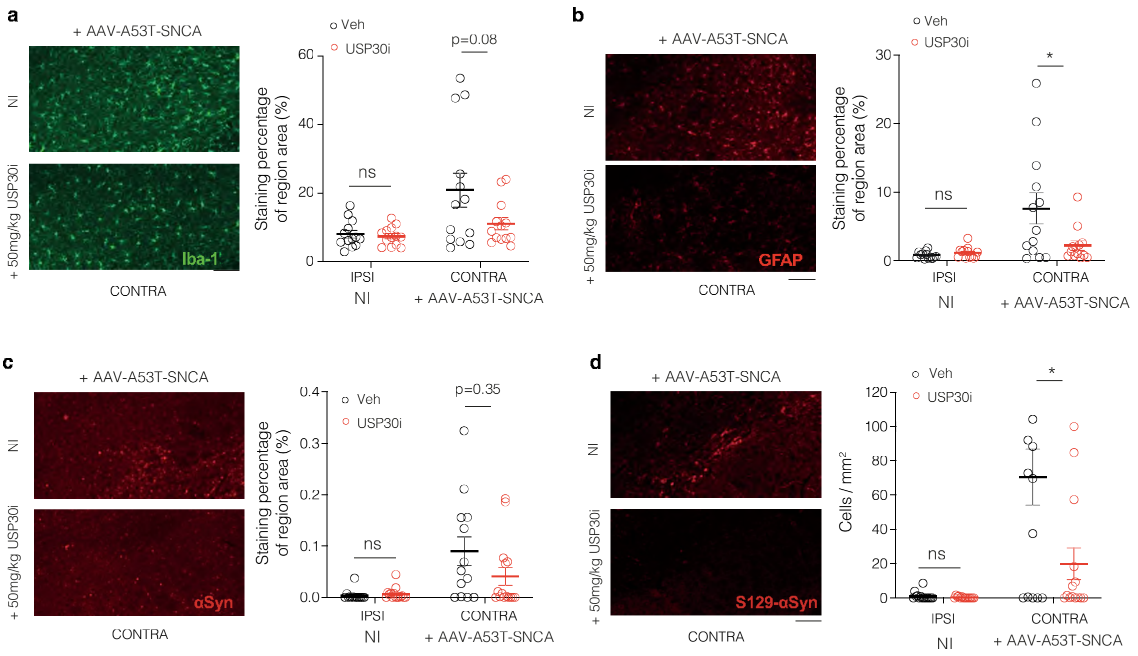

Supplementary Figure 8. Uncropped histology images used in the paper.

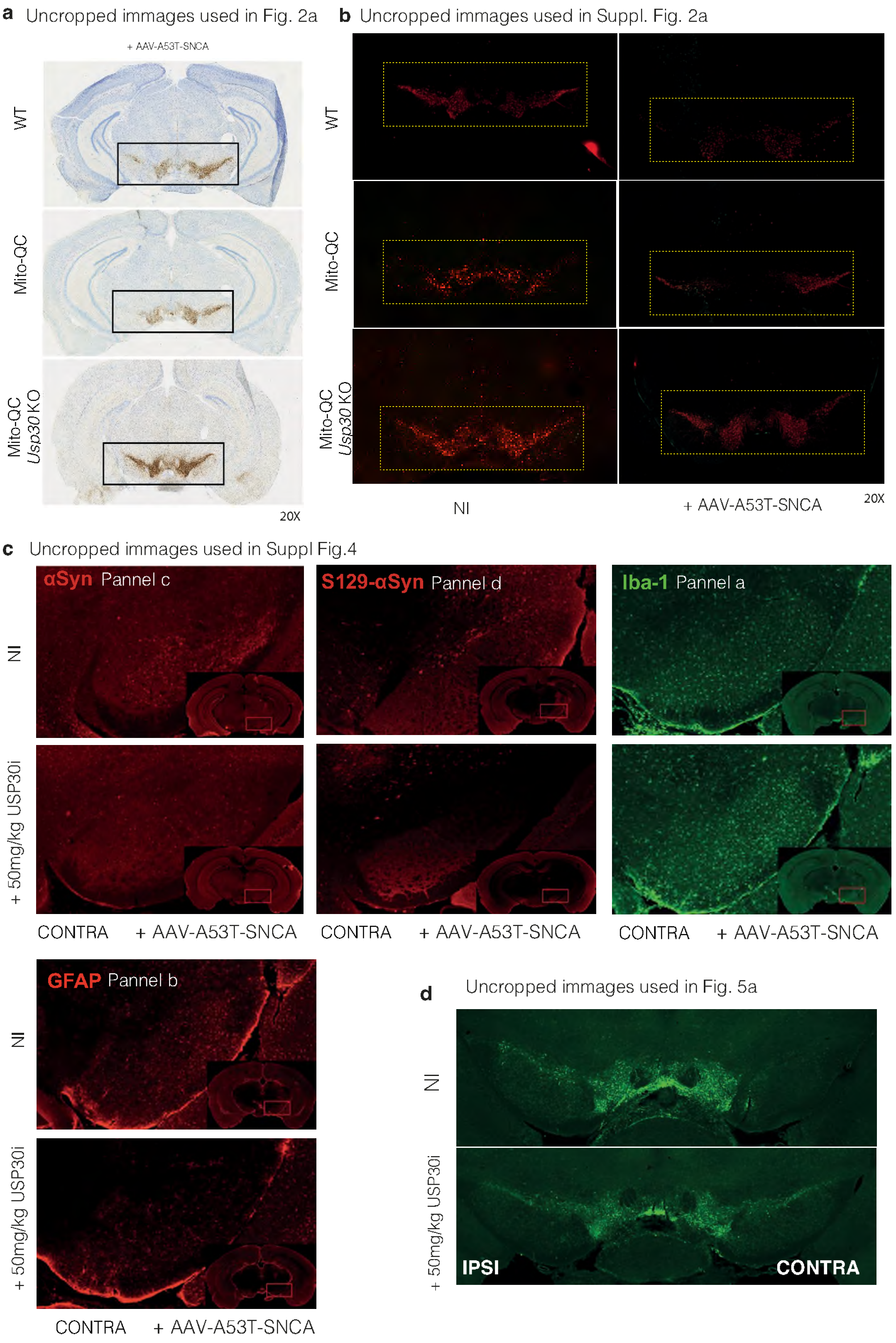

Supplementary Figure 9. Uncropped IF images used in the paper.

Uncropped IF immages used in Fig. 4e

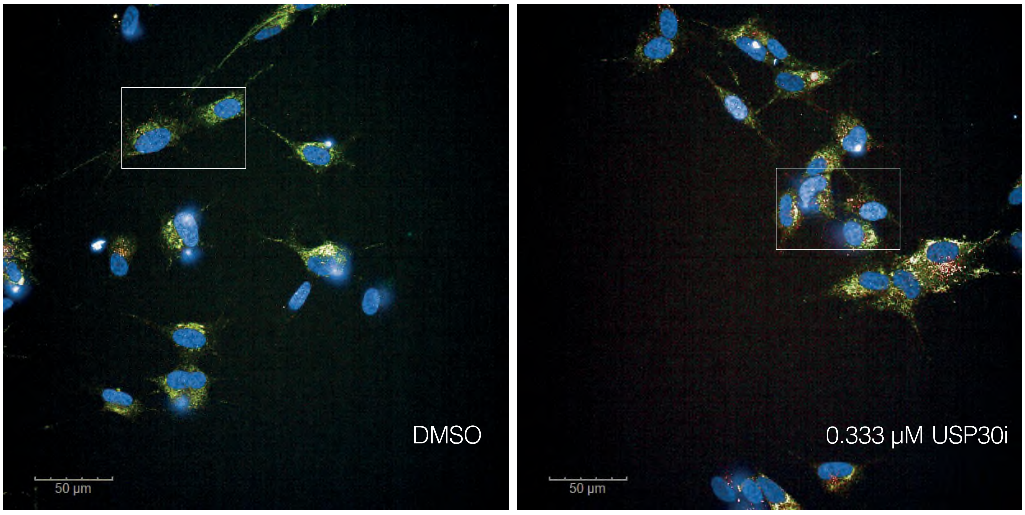

Mito-QC SH-SY5Y + 0.1μM A/O
