# Supplementary material for: Knockout or inhibition of USP30 protects dopaminergic neurons in a Parkinson’s disease mouse model
Source: Nat Commun. 2023 Nov 13;14:7295. doi: 10.1038/s41467-023-42876-1 (PMC10643470; doi:10.1038/s41467-023-42876-1)
Supplement: Supplementary file 3 — Description of Additional Supplementary Files [file 41467_2023_42876_MOESM3_ESM.docx]

**Description of Additional Supplementary Files**

**Supplementary Video 1:** Cylinder test of WT mice at 28 weeks after AAV-A53TSNCA injection.

**Supplementary Video 2:** Cylinder test of mito-QC mice at 28 weeks after AAV-A53TSNCA injection.

**Supplementary Video 3:** Cylinder test of mitoQC/USP30 KO mice at 28 weeks after AAV-A53T-SNCA injection.

**Supplementary Data 1.** Phenotypic analysis of Usp30 KO mice. Phenotypic analyses were performed on 709 mice (n=348 WT males; n=348 WT females; n=7 Usp30 KO males and n=6 Usp30 KO females). They quantified over 300 parameters covering categorical (dysmorphology, eye morphology), continuous (body weight – BW; PBL; haematological – Haem.; glucose tolerance - GTT; clinical chemistry – Clin. Chem.; calorimetry – Calo.; grip strength; heart weight; X-Ray’s and bone density scan - DEXA) and auditory brain stem response (ABR). All data have been deposited on the International Mouse Phenotypic Consortium (IMPC).
