# Supplementary material for: Knockout or inhibition of USP30 protects dopaminergic neurons in a Parkinson’s disease mouse model
Source: Nat Commun. 2023 Nov 13;14:7295. doi: 10.1038/s41467-023-42876-1 (PMC10643470; doi:10.1038/s41467-023-42876-1)
Supplement: Supplementary file 8 — Reporting Summary [file 41467_2023_42876_MOESM8_ESM.pdf]

nature portfolio

Corresponding author(s): Tracy-Shi Zhang Fang; Paul W. Thompson; Gabriel Balmus

Last updated by author(s): Sep 28, 2023

Reporting Summary

Nature Portfolio wishes to improve the reproducibility of the work that we publish. This form provides structure for consistency and transparency in reporting. For further information on Nature Portfolio policies, see our Editorial Policies and the Editorial Policy Checklist.

Statistics

For all statistical analyses, confirm that the following items are present in the figure legend, table legend, main text, or Methods section.

n/a	Confirmed	
☐	☒	The exact sample size (*n*) for each experimental group/condition, given as a discrete number and unit of measurement
☒	☐	A statement on whether measurements were taken from distinct samples or whether the same sample was measured repeatedly
☐	☒	The statistical test(s) used AND whether they are one- or two-sided<br>*Only common tests should be described solely by name; describe more complex techniques in the Methods section.*
☐	☒	A description of all covariates tested
☐	☒	A description of any assumptions or corrections, such as tests of normality and adjustment for multiple comparisons
☐	☒	A full description of the statistical parameters including central tendency (e.g. means) or other basic estimates (e.g. regression coefficient) AND variation (e.g. standard deviation) or associated estimates of uncertainty (e.g. confidence intervals)
☐	☒	For null hypothesis testing, the test statistic (e.g. *F*, *t*, *r*) with confidence intervals, effect sizes, degrees of freedom and *P* value noted<br>*Give P values as exact values whenever suitable.*
☒	☐	For Bayesian analysis, information on the choice of priors and Markov chain Monte Carlo settings
☐	☒	For hierarchical and complex designs, identification of the appropriate level for tests and full reporting of outcomes
☒	☐	Estimates of effect sizes (e.g. Cohen's *d*, Pearson's *r*), indicating how they were calculated

Our web collection on statistics for biologists contains articles on many of the points above.

Software and code

Policy information about availability of computer code

Data collection	The QuPath v0.2.0 software ((https://qupath.github.io) was used for counting TH+ neurons in SNpc.<br>The ImageJ software (NIH) was used to quantify the densitometry of TH staining in the striatum, mitophagy puncta analysis and colocalization analysis and WesternBlot bands.
Data analysis	GraphPad Prism (Prism 9.3.0) software was used for data analysis.

For manuscripts utilizing custom algorithms or software that are central to the research but not yet described in published literature, software must be made available to editors and reviewers. We strongly encourage code deposition in a community repository (e.g. GitHub). See the Nature Portfolio guidelines for submitting code & software for further information.

Data

Policy information about availability of data

All manuscripts must include a data availability statement. This statement should provide the following information, where applicable:

- Accession codes, unique identifiers, or web links for publicly available datasets
- A description of any restrictions on data availability
- For clinical datasets or third party data, please ensure that the statement adheres to our policy

The primary datasets have been uploaded with the manuscript. All datasets were collected by the authors listed in the manuscript.

Human research participants

Policy information about studies involving human research participants and Sex and Gender in Research.

Reporting on sex and gender	N/A
Population characteristics	N/A
Recruitment	N/A
Ethics oversight	N/A

Note that full information on the approval of the study protocol must also be provided in the manuscript.

Field-specific reporting

Please select the one below that is the best fit for your research. If you are not sure, read the appropriate sections before making your selection.

☒ Life sciences ☐ Behavioural & social sciences ☐ Ecological, evolutionary & environmental sciences

For a reference copy of the document with all sections, see nature.com/documents/nr-reporting-summary-flat.pdf

Life sciences study design

All studies must disclose on these points even when the disclosure is negative.

Sample size	For the USP30 KO study: In order to perform the required assessments, 2 sets of mice will be required: 1 for analyses requiring fixed tissue (immunohistochemistry) and 1 for analyses requiring fresh frozen tissue (Western blots, qPCR). Prior experience with these methods (PMID: 23571845; PMID: 17937603) indicates that an n=8 per experimental group per set provides sufficient power for each of these methods, except for the behavioral analyses which require an n=16. Both sets of mice per experimental group can be used for behavioral analyses prior to sacrifice, providing an n=16. We also included both female and male mice according to NIH policy. The total number for the KO study is 16 mice x 3 experimental groups x 2 AAV vectors x 2 sex=192 mice.
Data exclusions	We excluded a few mice in some of the groups, because the mice died before the ending point of the study.
Replication	We have enough n for each statistical analysis. Each data dots were collected from an individual mouse. We also have both female and male mice included for the the biological variable concern based on NIH policy. We reproduced the behavioral deficits and pathological alpha-syuclein in the PD model in both our pilot study and the real experiments. Each data was generated from replicates of 3 at least.
Randomization	All the mice were randomly assigned to experimental groups using free randomization tool, https://www.randomizer.org/
Blinding	The data were collected and anlyzed by investigators who were blinded to the treatments.

Reporting for specific materials, systems and methods

We require information from authors about some types of materials, experimental systems and methods used in many studies. Here, indicate whether each material, system or method listed is relevant to your study. If you are not sure if a list item applies to your research, read the appropriate section before selecting a response.

Materials & experimental systems

n/a	Involved in the study	
☐	☒	Antibodies
☐	☒	Eukaryotic cell lines
☒	☐	Palaeontology and archaeology
☐	☒	Animals and other organisms
☒	☐	Clinical data
☒	☐	Dual use research of concern

Methods

n/a	Involved in the study	
☒	☐	ChIP-seq
☒	☐	Flow cytometry
☒	☐	MRI-based neuroimaging

Antibodies

Antibodies used	anti-GFP (Aves Labs Inc, catalog # GFP-1010, lot # GFP3717982, 1:500 dilution), anti-mCherry (EMDmillipore, catalog #AB356482, lot # 3249537,1:500 dilution), anti-TH (KO studies: EMDmillipore, catalog # AB152, lot # 3870479, 1:1000 dilution; compound study:

Abcam, #ab76442, 1:1000 dilution), anti-USP30 (Santa Cruz Biotechnology, clone B-6, catalog # sc-515235, lot # C2620, 1:1000 dilution, validation of USP30 KO; MRC PPU Reagents and Services, #S746D, lot # 4, 1:600 dilution, cellular ubiquitin probe binding assay), anti-LAMP1 (Invitrogen, Clone eBio1D4B, catalog #14-1071-82, lot # 2162716, 1:500 dilution), anti-alpha-synuclein (phospho S129) antibody (Abcam, clone 81A, catalog # ab184674, lot # GR3407805-1, 1:1000 dilution), anti-Alpha-synuclein (phospho S129) antibody (Abcam, clone EP1536Y, catalog # ab51253, lot # GR3437967-8, 1:1000 dilution), anti-α-Synuclein (human) monoclonal antibody (15G7) (ENZO, clone 15G7, catalog # ALX-804-258, lot # 12071802,1:500 dilution), anti-OPA-1 (BD Transduction Laboratories, clone 18, catalog # 612606, lot # 8066752, 1:1000 dilution), anti-beta-actin (Santa Cruz Biotechnology, clone 2A3, catalog # sc-517582, lot # A1118,1:1000 dilution), anti-TOM20 (D8T4N) (Cell Signalling, clone D8T4N, catalog #42406, lot # 4,1:1000 dilution), Alexa Fluor secondary antibodies (1:500 dilution) were from Invitrogen, HRP-conjugated secondary antibodies (1:1000 dilution) were from Cell signaling technology, Biotinylated secondary antibody (1:1000 dilution) from Vector Laboratories.

Validation

Anti-GFP antibody was used for immunofluorescence (IF) on mouse brain sections. Wildtype mouse brains sections were used as negative control for validation of GFP expression in mito-QC mouse brain. The anti-GFP antibody was validated by the manufacturer in transgenic mice expressing the GFP gene product (https://www.aveslabs.com/products/anti-green-fluorescent-protein-antibody-gfp).
Anti-mCherry antibody was used for IF on mouse brain sections. Wildtype mouse brains sections were used as negative control for validation of mCherry expression in mito-QC mouse brain. The antibody was validated by the manufacturer for use in IF (https://www.emdmillipore.com/US/en/product/Anti-mCherry,MM_NF-AB356482).
Anti-TH antibody was used for IF on mouse brain sections. We validated the antibody with brain sections of substantia nigra. The antibody was validated by the manufacturer for use in IF (https://www.emdmillipore.com/US/en/product/Anti-Tyrosine-Hydroxylase-Antibody,MM_NF-AB152).
Anti-USP30 antibody was used for Western Blot (WB) in the knockout study. We validated the antibody with brain lysates from USP30 WT and USP30 KO mice. The antibody was validated by the manufacturer for use in WB (https://www.scbt.com/p/usp30-antibody-b-6).
Anti-LAMP1 antibody was used for IF in the knockout study. We validated the antibody with mouse brain sections. The antibody was validated in mouse thioglycolate-elicited peritoneal exudate cells by the manufacturer for the use in IF (https://www.thermofisher.com/antibody/product/CD107a-LAMP-1-Antibody-clone-eBio1D4B-1D4B-Monoclonal/14-1071-82).
Anti-alpha-synuclein (phosphor S129) antibody was used for IF in mouse brain sections. We validated the antibody with mouse brain sections with or without human A53T alpha-synulcein overexpression. The antibody was validated by the manufacturer for use in IHC (https://www.abcam.com/alpha-synuclein-phospho-s129-antibody-p-syn81a-ab184674.html).
Anti-OPA-1 antibody was used for WB in mouse brain samples. We validated the antibody with mouse brain lysates. The antibody was validated by the manufacturer for use in WB (https://www.bdbiosciences.com/en-us/products/reagents/microscopy-imaging-reagents/immunofluorescence-reagents/purified-mouse-anti-opa1.612606).
Anti-actin antibody was used for WB in knockout study. We validated the antibody with mouse brain lysates. The antibody was validated by the manufacturer for detecting beta-actin in mouse sample by WB (https://www.scbt.com/p/beta-actin-antibody-2a3).

Eukaryotic cell lines

Policy information about cell lines and Sex and Gender in Research

Cell line source(s)

State the source of each cell line used and the sex of all primary cell lines and cells derived from human participants or vertebrate models.

Authentication

Describe the authentication procedures for each cell line used OR declare that none of the cell lines used were authenticated.

Mycoplasma contamination

Confirm that all cell lines tested negative for mycoplasma contamination OR describe the results of the testing for mycoplasma contamination OR declare that the cell lines were not tested for mycoplasma contamination.

Commonly misidentified lines
(See ICLAC register)

Name any commonly misidentified cell lines used in the study and provide a rationale for their use.

Animals and other research organisms

Policy information about studies involving animals; ARRIVE guidelines recommended for reporting animal research, and Sex and Gender in Research

Laboratory animals

In USP30 Knockout study, we used mice on C57BL/6N genetic background from age 12 weeks to 40 weeks old. For mitophagy signal quantification in mito-QC mice, the mice were 16 weeks old.

Wild animals

No wild animals were used in the study.

Reporting on sex

We included both female (84) and male (86) mice in the knockout study according to the NIH policy. We collected behavioral assessment and monoamine analysis data from all mice and other data only in male mice. We did not find sex variability in our study.

Field-collected samples

No field-collected samples were used in the study.

Ethics oversight	Mouse studies at BIDMC were approved by the local Institutional Animal Care and Use Committee (IACUC). At Charles River Finland laboratories, the mouse work was done in accordance with all applicable national, international, and/or institutional guidelines for the care and use of animals (Finnish national legislation: 1) Act on the Protection of Used for Scientific or Educational Purposes (497/2013) 2) Government Decree on the Protection of Animals Used for Scientific or Educational Purposes (564/2013) 3) License for animal experiment approved by National Animal Experiment Board: 18537-2018. European and international legislation and guidelines: 1) Directive 2010/63/EU 2) Commission recommendation 2007/526/EC 3) Guide for the Care and Use of Laboratory Animals (Guide), Eighth Edition (National Research Council 2011)).

Note that full information on the approval of the study protocol must also be provided in the manuscript.
